# FOXO1 suppresses PGC‐1β gene expression in skeletal muscles

**DOI:** 10.1002/2211-5463.12898

**Published:** 2020-07-01

**Authors:** Shiho Nakai, Mamoru Oyabu, Yukino Hatazawa, Shiori Akashi, Tadahiro Kitamura, Shinji Miura, Yasutomi Kamei

**Affiliations:** ^1^ Graduate School of Life and Environmental Sciences Kyoto Prefectural University Kyoto Japan; ^2^ Graduate School of Nutritional and Environmental Sciences University of Shizuoka Shizuoka Japan; ^3^ Metabolic Signal Research Center Institute for Molecular and Cellular Regulation Gunma University Gunma Japan

**Keywords:** atrophy, FOXO1, PGC‐1β, skeletal muscle, transcriptional factor

## Abstract

Peroxisome proliferator‐activated receptor‐gamma coactivator‐1β (PGC‐1β) is a transcriptional regulator whose increased expression activates energy expenditure‐related genes in skeletal muscles. However, how PGC‐1β is regulated remains largely unclear. Here, we show that PGC‐1β gene expression is negatively correlated with the expression of a transcription factor, forkhead box protein O1 (FOXO1), whose expression is increased during muscle atrophy. In the skeletal muscles of FOXO1‐overexpressing transgenic mice, PGC‐1β gene expression is decreased. Denervation or plaster cast‐based unloading, as well as fasting, increases endogenous FOXO1 expression in skeletal muscles, with decreased PGC‐1β expression. In the skeletal muscles of FOXO1‐knockout mice, the decrease in PGC‐1β expression caused by fasting was attenuated. Tamoxifen‐inducible FOXO1 activation in C2C12 myoblasts causes a marked decrease of PGC‐1β expression. These findings together reveal that FOXO1 activation suppresses PGC‐1β expression. During atrophy with FOXO1 activation, decreased PGC‐1β may decrease energy expenditure and avoid wasting energy.

## Graphical abstract

Gene expression of PGC‐1β (energy expenditure regulator) is negatively regulated by forkhead box protein O1 (FOXO1, atrophy regulator). Denervation, plaster cast‐based unloading, or fasting increased FOXO1 expression in skeletal muscles, with decreased PGC‐1β expression. Gain or loss of function of FOXO1 revealed that FOXO1 activation suppresses PGC‐1β expression. During atrophy with FOXO1 activation, decreased PGC‐1β may lead to decreased energy expenditure, avoiding wasting energy.

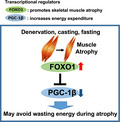

**Abbreviations**
ER: estrogen receptor
FOXO1: forkhead box protein O1
MCAD: medium‐chain acyl CoA dehydrogenase
PGC‐1β: peroxisome proliferator‐activated receptor‐gamma coactivator‐1β
Tg: transgenic

FOXO1 (Gene symbol; *Foxo1*) is a forkhead‐type transcription factor, whose expression is markedly upregulated in skeletal muscles during atrophy, that is, under conditions such as starvation, unloading (plaster cast), and denervation [1, 2]. Transgenic (Tg) overexpression of FOXO1 in skeletal muscles causes muscle atrophy [3], with increased expression of atrophy‐related genes, including cathepsin L (*Ctsl*) and lysosomal proteinase [3, 4]. In the skeletal muscles of FOXO1‐knockout (FOXO1‐KO) mice, the increase in cathepsin L gene expression caused by fasting was attenuated [4, 5]. FOXO1 activation mostly increases the expression of its target genes [6, 7]; however, the expression of some genes such as IGFBP5 (*Igfbp5*) and musclin (or osteocrin, *Ostn*) is decreased by FOXO1 [3, 8].

Peroxisome proliferator‐activated receptor‐gamma coactivator‐1β (PGC‐1β; *Ppargc1b*) is a transcriptional coactivator of nuclear receptors, which is a homolog of PGC‐1α (*Ppargc1a*) [9, 10]. Both PGC‐1β and PGC‐1α are known to increase the mitochondrial content in cells [11, 12]. PGC‐1β and PGC‐1α activate nuclear receptors, such as the estrogen‐related receptor [10], and activate target genes (i.e., medium‐chain acyl CoA dehydrogenase, MCAD, *Acadm*) in skeletal muscles [10, 13, 14]. Indeed, the overexpression of PGC‐1β in skeletal muscles in mice led to increased energy expenditure and an anti‐obesity phenotype [10]. Regulation of PGC‐1α in skeletal muscles has been well studied. PGC‐1α expression is markedly upregulated during exercise [15, 16] and is considered to contribute to the expression of exercise‐related genes, such as those involved in branched‐chain amino acid metabolism [17]. In contrast, little is known about the regulation of the PGC‐1β gene in skeletal muscles.

In this study, we attempted to analyze the possible FOXO1‐mediated PGC‐1β gene expression, as the level of PGC‐1β mRNA was decreased in the skeletal muscles of FOXO1‐overexpressing Tg mice. Thus, we examined the level of PGC‐1β gene expression in various conditions with altered FOXO1 levels in skeletal muscles and cells.

## Materials and methods

### Animals

Tg mice overexpressing FOXO1 in skeletal muscles (FOXO1‐Tg) have been previously described [3]. Skeletal muscle‐specific FOXO1‐KO mice were described previously [5]. C57BL/6J mice were purchased from Shimizu Laboratory Supplies Co., Ltd. (Kyoto, Japan) and maintained at a constant temperature (24 °C) with fixed artificial light (12‐h light/12‐h dark cycle). All animal experiments were performed in accordance with the guidelines of the Kyoto Prefectural University Committee on Animal Research. The protocol was approved by this committee (no. KPU260407, review board: Y. Tsukamoto).

### cDNA microarray analysis

RNA was isolated from skeletal muscle (gastrocnemius) of FOXO1‐Tg mice (age, 25 weeks) and age‐matched wild‐type control mice. Samples from wild‐type (*N* = 6) and FOXO1‐Tg mice (*N* = 5) were pooled and used. RNA was isolated using TRIzol reagent (Thermo Fisher Scientific Inc., Tokyo, Japan) and purified using an RNeasy Mini kit (Qiagen, Hilden, Germany). Each sample was labeled with cyanine 3‐CTP using a Low Input Quick Amp Labeling Kit (Agilent Technologies, Santa Clara, CA, USA). Cyanine 3‐CTP‐labeled cRNA (1.65 μg) was fragmented and hybridized to the Agilent whole mouse genome (8 × 60 K) microarray. Signal detection and data analysis were performed as described previously [17]. The microarray data were submitted to the Gene Expression Omnibus (GEO) database (https://www.ncbi.nlm.nih.gov/geo/). The records have been assigned GEO accession numbers as GSE146919.

### Quantitative real‐time RT‐PCR analysis

Total RNA was isolated from skeletal muscles or cells using TRIzol reagent (Thermo Fisher Scientific Inc.). cDNA was synthesized using 500 ng of each RNA sample with ReverTraAce (Toyobo, Tokyo, Japan). Gene expression was measured as described previously [18]. Fold change for each target gene was calculated as follows: ΔC*_t_* = C*_t_* (target gene) – C*_t_* (reference gene), ΔΔC*_t_* = ΔC*_t_* (target gene) – ΔC*_t_* (reference gene). Due to the exponential nature of PCR, ‘fold change’ was calculated as $2^{-\Delta \Delta C_{t}}$ [19]. The primer sequences used were as follows: FOXO1, forward 5′‐GCGGGCTGGAAGAATTCAAT‐3′ and reverse 5′‐TCCAGTTCCTTCATTCTGCA‐3′; cathepsin L, forward 5′‐TCTCACGCTCAAGGCAATCA‐3′ and reverse 5′‐AAGCAAAATCCATCAGGCCTC‐3′; PGC‐1β, forward 5′‐AGAGGCACCCAGAGCGAAG‐3′ and reverse 5′‐TTGTGGCATGCTGCAAATG‐3′; MCAD, forward 5′‐GATCGCAATGGGTGCTTTTGATAGAA‐3′ and reverse 5′‐AGCTGATTGGCAATGTCTCCAGCAAA‐3′; PGC‐1α, forward 5′‐CGGAAATCATATCCAACCAG‐3′ and reverse 5′‐TGAGGACCGCTAGCAAGTTTG‐3′; MyoD, forward 5′‐ CGGGACATAGACTTGACAGGC‐3′ and reverse 5′‐ TCGAAACACGGGTCATCATAGA‐3′; myogenin, forward 5′‐ CATGGTGCCCAGTGAATGCAACTC‐3′ and reverse 5′‐ TATCCTCCACCGTGATGCTGTCCA‐3′; 36B4, forward 5′‐GGCCCTGCACTCTCGCTTTC‐3′ and reverse 5′‐TGCCAGGACGCGCTTGT‐3′, and 18S, forward 5′‐GGGAGCCTGAGAAACGGC‐3′ and reverse 5′‐ GGGTCGGGAGTGGGTAATTTT‐3.

### Western blotting analysis

Western blotting analysis was performed as described previously [5]. The primary antibody used was anti‐FOXO1 [FoxO1 (C29H4) Rabbit mAb #2880; Cell Signaling Technology, Danvers, MA, USA].

### Measurement of mitochondrial DNA content

Mitochondrial DNA (mtDNA) content was measured as mtDNA copy number normalized to the copy number of a gene contained in the nuclear genome. The mitochondrial gene used for mtDNA copy estimation was cytochrome c oxidase subunit 2 (COX2), and the copy number of COX2 was normalized to the copy number of the 36B4 gene, contained in the nuclear genome, as described previously [20].

### Measurement of citrate synthase activity

Citrate synthase (CS) activity was measured as described previously [21].

### Denervation, plaster cast, and fasting

For the denervation model, a 4‐ to 5‐mm section of the sciatic nerve in the hindlimb of the mice was removed [18]. After 12 days, skeletal muscles were collected.

A plaster cast for the mice was created as described previously [18]. The hindlimb skeletal muscles of the mice were immobilized (unloaded) by the plaster cast. After 11 days, skeletal muscles were collected.

For the fasting experiment, C57BL/6J mice (9 weeks old, male) were fasted for 8 or 24 h. For refeeding, the mice were fasted for 24 h and refed for 4 h. Then, skeletal muscles were collected [22].

### Cells

C2C12 mouse myoblasts (Riken Cell Bank, Tsukuba, Japan) stably expressing the FOXO1‐estrogen receptor (ER) fusion protein were prepared as previously described [4, 23, 24]. In brief, C2C12 cells were stably transfected with the pBABE retroviral vector expressing fusion proteins containing a constitutively active form of human FOXO1, in which the AKT phosphorylation sites Thr‐24, Ser‐256, and Ser‐319 are replaced with alanine [FOXO1(3A)] in‐frame with a modified tamoxifen‐specific version of the ligand‐binding domain murine ER [4, 23]. Fusion proteins were restricted to the cytoplasmic compartment until activation with tamoxifen, which caused FOXO1‐ER to relocate to the nucleus, where the FOXO1 moiety then functioned as a transcription factor [4, 23]. The cells were then cultured in Dulbecco’s modified Eagle’s medium supplemented with 10% FBS. The medium was replaced every 2 days until the cells reached confluence. Two days after confluence, the cells (undifferentiated myoblasts) were treated with tamoxifen for 24 h and used for the RNA analysis.

### Statistical analyses

Statistical analyses were performed using Student’s two‐tailed unpaired *t*‐test for comparisons between two groups, and one‐way analysis of variance followed by Tukey’s *post hoc* test for comparisons between three or more groups. Two‐way analysis of variance followed by Tukey’s *post hoc* test for FOXO1‐KO mice analysis. *P* < 0.05 was considered significant.

## Results and Discussion

### Decreased PGC‐1β expression in the skeletal muscles of FOXO1‐Tg mice

**Fig. 1:**
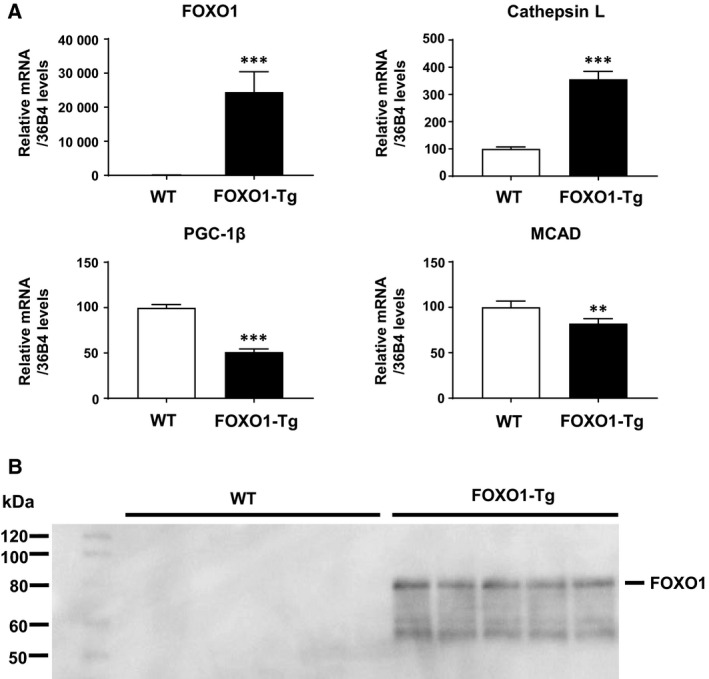
Gene expression analysis of FOXO1, cathepsin L, PGC‐1β, and MCAD in the skeletal muscles of FOXO1‐overexpressing mice. (A) FOXO1 was remarkably expressed in FOXO1‐Tg mice. Cathepsin L, the target gene of FOXO1, was also increased in FOXO1‐Tg mice. In contrast, the expression of PGC‐1β and MCAD decreased in FOXO1‐Tg mice. Quantitative real‐time RT‐PCR data from wild‐type (WT) control mice were set at 100 arbitrary units. Each value is presented as the mean ± standard error (SE; *N* = 5). Statistical analyses were performed using Student’s two‐tailed unpaired *t*‐test. ***P < 0.001, **P < 0.01 versus wild‐type. (B) Western blotting analysis of skeletal muscle from FOXO1‐Tg mice.

First, we used a skeletal muscle sample of FOXO1‐overexpressing Tg (FOXO1‐Tg) mice [3]. The skeletal muscle weight of the wild‐type control was 190 ± 9 mg (*N* = 5) and that of the FOXO1‐Tg mice was 117 ± 7 mg (*N* = 5; *P* < 0.001), reflecting muscle atrophy in the latter group. We performed microarray analysis to understand the gene expression changes caused by FOXO1 overexpression. One‐hundred‐and‐fifty‐three genes were upregulated more than twofold, and 145 genes were downregulated more than 0.5‐fold (Tables 1 and 2). Microarray data showed decreased PGC‐1β expression in the skeletal muscles of FOXO1‐Tg mice, compared with that in wild‐type control mice (0.44‐fold; Table 2). In order to confirm the microarray data, we examined the gene expression using real‐time qPCR. As expected, FOXO1 transgene overexpression was observed in the skeletal muscles of the FOXO1‐Tg mice (Fig. 1A). We observed increased FOXO1 protein levels in the skeletal muscle of FOXO1‐Tg mice (Fig. 1B). Authentic FOXO1 target gene cathepsin L expression was markedly increased in the FOXO1‐Tg mice (Fig. 1A), indicating the functional expression of the FOXO1 transgene. At the same time, PGC‐1β gene expression was significantly decreased in the FOXO1‐Tg mice (Fig. 1A), confirming the microarray data. In addition, the expression of the known PGC‐1β target MCAD was significantly decreased. Thus, FOXO1 overexpression appears to decrease PGC‐1β expression in skeletal muscles.

**Table 1:** List of genes in skeletal muscle with increased expression levels in FOXO1‐Tg mice compared with wild‐type control mice. Top 100 genes are shown.

	SystematicName	GeneName	Description	Fold (FOXO1‐Tg/ Wild‐type)
1	NM_025540	Sln	Sarcolipin	154.62
2	NM_001081187	Htra4	HtrA serine peptidase 4	66.04
3	NM_019739	Foxo1	Forkhead box O1	56.54
4	NM_010858	Myl4	Myosin, light polypeptide 4	14.98
5	NM_001134697	Ctxn3	Cortexin 3	11.60
6	NM_013803	Casr	Calcium‐sensing receptor	9.63
7	NM_007836	Gadd45a	Growth arrest and DNA‐damage‐inducible 45 alpha	7.61
8	NM_013492	Clu	Clusterin	7.22
9	NM_025359	Tspan13	Tetraspanin 13	7.18
10	NM_030695	Lrba	LPS‐responsive beige‐like anchor	6.99
11	NM_010597	Kcnab1	Potassium voltage‐gated channel, shaker‐related subfamily, beta member 1	5.78
12	NM_146085	Apbb3	Amyloid beta (A4) precursor protein‐binding, family B, member 3	5.16
13	NM_008362	Il1r1	Interleukin 1 receptor, type I	5.07
14	NM_007913	Egr1	Early growth response 1	4.71
15	NM_153578	Nipa1	Nonimprinted in Prader‐Willi/Angelman syndrome 1 homolog (human)	4.62
16	NM_011044	Pck1	Phosphoenolpyruvate carboxykinase 1, cytosolic	4.46
17	NM_008258	Hn1	Hematological and neurological expressed sequence 1	4.36
18	NM_201256	Eif4ebp3	Eukaryotic translation initiation factor 4E binding protein 3	4.28
19	NM_001102405	Acp5	Acid phosphatase 5, tartrate resistant	4.15
20	NM_021282	Cyp2e1	Cytochrome P450, family 2, subfamily e, polypeptide 1	4.15
21	NM_009876	Cdkn1c	Cyclin‐dependent kinase inhibitor 1C (P57)	4.15
22	NM_021282	Cyp2e1	Cytochrome P450, family 2, subfamily e, polypeptide 1	4.14
23	NM_025439	Tmem9	Transmembrane protein 9	4.12
24	NM_144936	Tmem45b	Transmembrane protein 45b	4.01
25	NM_008086	Gas1	Growth arrest‐specific 1	4.00
26	NM_013614	Odc1	Ornithine decarboxylase, structural 1	3.90
27	NM_011858	Tenm4	Teneurin transmembrane protein 4	3.87
28	NM_001204959	Retn	Resistin	3.84
29	NM_178373	Cidec	Cell death‐inducing DFFA‐like effector c	3.76
30	NM_009605	Adipoq	Adiponectin, C1Q, and collagen domain containing	3.69
31	NM_008161	Gpx3	Glutathione peroxidase 3	3.66
32	NM_007389	Chrna1	Cholinergic receptor, nicotinic, alpha polypeptide 1 (muscle)	3.55
33	NM_025869	Dusp26	Dual specificity phosphatase 26 (putative)	3.50
34	NM_011158	Prkar2b	Protein kinase, cAMP‐dependent regulatory, type II beta	3.44
35	NM_175640	Plin1	Perilipin 1	3.42
36	NM_001159487	Rbp4	Retinol binding protein 4, plasma	3.37
37	NM_033037	Cdo1	Cysteine dioxygenase 1, cytosolic	3.37
38	NM_026929	Chac1	ChaC, cation transport regulator 1	3.37
39	NM_181072	Myo1e	Myosin IE	3.35
40	NM_013459	Cfd	Complement factor D (adipsin)	3.34
41	NM_029385	Nudt16	Nudix (nucleoside diphosphate linked moiety X)‐type motif 16	3.34
42	NM_009675	Aoc3	Amine oxidase, copper containing 3	3.32
43	NM_009127	Scd1	Stearoyl‐Coenzyme A desaturase 1	3.30
44	NM_007469	Apoc1	Apolipoprotein C‐I	3.27
45	NM_177733	E2f2	E2F transcription factor 2	3.25
46	NM_013869	Tnfrsf19	Tumor necrosis factor receptor superfamily, member 19	3.23
47	NM_010864	Myo5a	Myosin VA	3.21
48	NM_029803	Ifi27l2a	Interferon, alpha‐inducible protein 27 like 2A	3.19
49	NM_010828	Cited2	Cbp/p300‐interacting transactivator, with Glu/Asp‐rich carboxy‐terminal domain, 2	3.14
50	NM_017370	Hp	Haptoglobin	3.13
51	NM_145400	Ube4a	Ubiquitination factor E4A, UFD2 homolog (*S. cerevisiae*)	3.06
52	NM_133838	Ehd4	EH‐domain containing 4	3.05
53	NM_007639	Cd1d1	CD1d1 antigen	3.05
54	NM_001013826	Dupd1	Dual specificity phosphatase and pro isomerase domain containing 1	2.95
55	NM_023625	Plbd2	Phospholipase B domain containing 2	2.95
56	NM_013822	Jag1	Jagged 1	2.93
57	NM_177409	Tram2	Translocating chain‐associating membrane protein 2	2.90
58	NM_020581	Angptl4	Angiopoietin‐like 4	2.89
59	NM_009822	Runx1t1	Runt‐related transcription factor 1; translocated to, 1 (cyclin D‐related)	2.89
60	NM_146001	Hip1	Huntingtin‐interacting protein 1	2.89
61	NM_011430	Sncg	Synuclein, gamma	2.89
62	NM_007679	Cebpd	CCAAT/enhancer binding protein (C/EBP), delta	2.88
63	NM_011580	Thbs1	Thrombospondin 1	2.85
64	NM_008630	Mt2	Metallothionein 2	2.84
65	NM_133955	Rhou	Ras homolog gene family, member U	2.83
66	NM_025888	Kctd20	Potassium channel tetramerization domain containing 20	2.82
67	NM_008198	Cfb	Complement factor B	2.81
68	NM_019432	Tmem37	Transmembrane protein 37	2.71
69	NM_013468	Ankrd1	Ankyrin repeat domain 1 (cardiac muscle)	2.71
70	NM_025593	Polr2l	Polymerase (RNA) II (DNA directed) polypeptide L	2.70
71	NM_001198823	App	Amyloid beta (A4) precursor protein (App)	2.69
72	NM_178087	Pml	Promyelocytic leukemia	2.68
73	NM_138673	Stab2	Stabilin 2	2.66
74	NM_007569	Btg1	B‐cell translocation gene 1, antiproliferative	2.66
75	NM_009984	Ctsl	Cathepsin L	2.63
76	NM_009801	Car2	Carbonic anhydrase 2	2.63
77	NM_008055	Fzd4	Frizzled homolog 4 (Drosophila)	2.60
78	ENSMUST00000030257	Cachd1	Cache domain containing 1	2.58
79	NM_146251	Pnpla7	Patatin‐like phospholipase domain containing 7	2.58
80	NM_197986	Tmem140	Transmembrane protein 140	2.58
81	NM_001198984	Tcof1	Treacher Collins Franceschetti syndrome 1, homolog	2.58
82	NM_009201	Slc1a5	Solute carrier family 1 (neutral amino acid transporter), member 5	2.54
83	NM_001145953	Lgals3	Lectin, galactose binding, soluble 3	2.52
84	NM_133977	Trf	Transferrin	2.50
85	NM_001081349	Slc43a1	Solute carrier family 43, member 1	2.50
86	NM_029083	Ddit4	DNA damage‐inducible transcript 4	2.50
87	NM_009780	C4b	Complement component 4B	2.50
88	NM_010097	Sparcl1	SPARC‐like 1	2.49
89	NM_001101433	Zcchc24	Zinc finger, CCHC domain containing 24	2.48
90	NM_133198	Pygl	Liver glycogen phosphorylase	2.44
91	NM_026439	Ccdc80	Coiled‐coil domain containing 80	2.44
92	NM_019412	Prx	Periaxin	2.41
93	NM_148927	Plekha4	Pleckstrin homology domain containing, family A	2.41
94	NM_181390	Mustn1	Musculoskeletal, embryonic nuclear protein 1	2.40
95	NM_001097644	Ccnyl1	Cyclin Y‐like 1	2.39
96	NM_026330	Nsmce1	Non‐SMC element 1 homolog (S. cerevisiae)	2.38
97	NM_008037	Fosl2	Fos‐like antigen 2	2.36
98	NM_001039386	Nsmf	NMDA receptor synaptonuclear signaling and neuronal migration factor	2.35
99	NM_023587	Ptplb	Protein tyrosine phosphatase‐like (proline instead of catalytic arginine), member b	2.32
100	NM_011785	Akt3	Thymoma viral proto‐oncogene 3	2.32

**Table 2:** List of genes in skeletal muscle with decreased expression levels in FOXO1‐Tg mice compared with wild‐type control mice. Top 100 genes are shown. PGC‐1β is highlighted.

	SystematicName	GeneName	Description	Fold (FOXO1‐Tg/ Wild‐type)
1	NM_010292	Gck	Glucokinase	0.06
2	NM_001081324	Neto2	Neuropilin (NRP) and tolloid (TLL)‐like 2	0.06
3	NM_001033473	Odf3l2	Outer dense fiber of sperm tails 3‐like 2	0.06
4	NM_198112	Ostn	Osteocrin	0.06
5	NM_011825	Grem2	Gremlin 2 homolog, cysteine knot superfamily (*Xenopus laevis*)	0.06
6	NM_009867	Cdh4	Cadherin 4	0.08
7	NM_053250	Crip3	Cysteine‐rich protein 3 (Crip3), transcript variant TLP‐B	0.09
8	NM_009700	Aqp4	Aquaporin 4	0.10
9	NM_177787	Slc15a5	Solute carrier family 15, member 5	0.12
10	NM_144547	Amhr2	Anti‐Mullerian hormone type 2 receptor	0.13
11	NM_013467	Aldh1a1	Aldehyde dehydrogenase family 1, subfamily A1	0.14
12	NM_030017	Rdh12	Retinol dehydrogenase 12	0.15
13	NM_011497	Aurka	Aurora kinase A	0.16
14	NM_001081160	Mdga1	MAM domain containing glycosylphosphatidylinositol anchor 1	0.16
15	NM_001013799	Mettl21c	Methyltransferase like 21C	0.16
16	NM_001024539	Shc2	SHC (Src homology 2 domain containing) transforming protein 2	0.17
17	NM_144860	Mib1	Mindbomb homolog 1 (Drosophila)	0.18
18	NM_176920	Lrtm1	Leucine‐rich repeats and transmembrane domains 1	0.18
19	NM_016749	Mybph	Myosin binding protein H	0.18
20	NM_010061	Dnase1	Deoxyribonuclease I	0.19
21	NM_029104	Mss51	MSS51 mitochondrial translational activator (Mss51), nuclear gene encoding mitochondrial protein	0.20
22	NM_001177841	Otub2	OTU domain, ubiquitin aldehyde binding 2	0.20
23	NM_010019	Dapk2	Death‐associated protein kinase 2	0.20
24	NM_025998	Nkain1	Na+/K+transporting ATPase interacting 1	0.20
25	NM_011943	Map2k6	Mitogen‐activated protein kinase kinase 6	0.22
26	NM_194060	Foxo6	Forkhead box O6	0.22
27	NM_028638	Gadl1	Glutamate decarboxylase‐like 1	0.22
28	NM_009393	Tnnc1	Troponin C, cardiac/slow skeletal	0.23
29	NM_145562	Parm1	Prostate androgen‐regulated mucin‐like protein 1	0.23
30	NM_010585	Itpr1	Inositol 1,4,5‐trisphosphate receptor 1	0.25
31	NM_001253822	Irx3	Iroquois‐related homeobox 3 (Drosophila)	0.26
32	NM_013737	Pla2g7	Phospholipase A2, group VII (platelet‐activating factor acetylhydrolase, plasma)	0.26
33	NM_019636	Tbc1d1	TBC1 domain family, member 1	0.26
34	NM_016719	Grb14	Growth factor receptor bound protein 14	0.27
36	NM_015814	Dkk3	dickkopf homolog 3 (*Xenopus laevis*)	0.28
37	NM_010267	Gdap1	Ganglioside‐induced differentiation‐associated‐protein 1	0.29
38	NM_022314	Tpm3	Tropomyosin 3, gamma	0.29
39	NM_007642	Cd28	CD28 antigen	0.29
40	NM_010246	Fzd9	frizzled homolog 9 (Drosophila)	0.29
41	NM_026999	Zfp688	Zinc finger protein 688	0.30
42	NM_031997	Tmem2	Transmembrane Protein 2	0.30
43	NM_016854	Ppp1r3c	Protein phosphatase 1, regulatory (inhibitor) subunit 3C	0.30
44	NM_001159344	Casz1	Castor zinc finger 1	0.30
45	NM_027402	Fndc5	Fibronectin type III domain containing 5	0.30
46	BC019757	Hist1h4i	Histone cluster 1, H4i	0.30
47	NM_010859	Myl3	Myosin, light polypeptide 3	0.30
48	NM_207161	Dnph1	2'‐deoxynucleoside 5'‐phosphate N‐hydrolase 1	0.31
49	NM_026884	Fam57b	Family with sequence similarity 57, member B	0.32
50	NM_010861	Myl2	Myosin, light polypeptide 2, regulatory, cardiac, slow	0.32
51	NM_010518	Igfbp5	Insulin‐like growth factor binding protein 5	0.32
52	NM_027963	Wdr16	WD repeat domain 16	0.32
53	NM_001081063	Prss55	Protease, serine, 55	0.32
54	NR_037996	Hmga2‐ps1	HIGH‐mobility group AT‐hook 2, pseudogene 1	0.32
55	NM_027161	Tmem52	Transmembrane protein 52	0.32
56	NM_019563	Cited4	Cbp/p300‐interacting transactivator, with Glu/Asp‐rich carboxy‐terminal domain, 4	0.33
57	NM_175511	Fam78a	Family with sequence similarity 78, member A	0.33
58	NM_175276	Fhod3	Formin homology 2 domain containing 3	0.34
59	NM_018760	Slc4a4	Solute carrier family 4 (anion exchanger), member 4	0.34
60	ENSMUST00000108587	Tnnt1	Troponin T1, skeletal, slow	0.35
61	NM_008852	Pitx3	Paired‐like homeodomain transcription factor 3	0.35
62	NM_080728	Myh7	Myosin, heavy polypeptide 7, cardiac muscle, beta	0.36
63	NM_018832	Magix	MAGI family member, X‐linked	0.37
64	NM_001170488	Tprkb	Tp53rk binding protein	0.37
65	NM_030241	Setd8	SET domain containing (lysine methyltransferase) 8	0.37
66	NM_007431	Alpl	Alkaline phosphatase, liver/bone/kidney	0.37
67	NM_181577	Ccdc85a	Coiled‐coil domain containing 85A	0.37
68	NM_001122683	Bdh1	3‐hydroxybutyrate dehydrogenase, type 1	0.37
69	NM_011983	Homer2	Homer homolog 2 (Drosophila)	0.37
70	NM_011638	Tfrc	Transferrin receptor	0.37
71	NM_030179	Clip4	CAP‐GLY domain containing linker protein family, member 4	0.37
72	NM_198190	Ntf5	Neurotrophin 5	0.37
73	NM_010834	Mstn	myostatin	0.38
74	NM_001085378	Myh7b	Myosin, heavy chain 7B, cardiac muscle, beta	0.38
75	NM_177603	Frat2	Frequently rearranged in advanced T cell lymphomas 2	0.38
76	NM_009519	Wnt11	Wingless‐related MMTV integration site 11	0.39
77	NM_133363	Myoz3	Myogenin 3	0.39
78	NM_027307	Golm1	Golgi membrane protein 1	0.39
79	NM_027678	Zranb3	Zinc finger, RAN‐binding domain containing 3	0.40
80	NM_001160262	Fam78b	Family with sequence similarity 78, member B	0.40
81	NM_148958	Osbpl10	Oxysterol binding protein‐like 10	0.40
82	EU616813	Mirg	Clone E19 5E_C11 maternally expressed gene 9	0.40
83	NM_021467	Tnni1	Troponin I, skeletal, slow 1	0.40
84	NR_003280	Rs5‐8s1	5.8S ribosomal RNA	0.41
85	NM_080595	Emid1	EMI domain containing 1	0.41
86	NM_001109040	Kif21a	Kinesin family member 21A	0.41
87	NM_033478	Ly6g6d	Lymphocyte antigen 6 complex, locus G6D	0.41
88	NM_173745	Dusp18	Dual specificity phosphatase 18	0.41
89	NM_018803	Syt10	Synaptotagmin X	0.41
90	NM_001252310	Fam19a5	Family with sequence similarity 19, member A5	0.41
91	NM_011160	Prkg1	Protein kinase, cGMP‐dependent, type I	0.42
92	NM_030263	Psd3	Pleckstrin and Sec7 domain containing 3	0.43
93	NM_010866	Myod1	Myogenic differentiation 1	0.43
94	NM_008421	Kcnc1	Potassium voltage‐gated channel, Shaw‐related subfamily, member 1	0.43
96	NM_009107	Rxrg	Retinoid X receptor gamma	0.44
97	NM_133249	Ppargc1b	Peroxisome proliferative activated receptor, gamma, coactivator 1 beta	0.44
98	NM_008596	Sypl2	Synaptophysin‐like 2	0.44
99	NM_001272024	Sema6c	Sema domain, transmembrane domain (TM), and cytoplasmic domain (semaphorin) 6C	0.44
100	NM_011103	Prkcd	Protein kinase C, delta	0.44

### Expression of PGC‐1β gene in skeletal muscles with changed endogenous FOXO1 expression

**Fig. 2:**
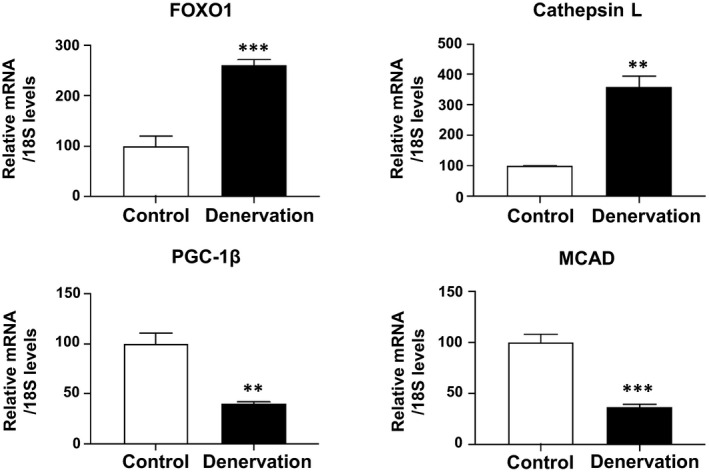
Gene expression analysis of FOXO1, cathepsin L, PGC‐1β, and MCAD in the skeletal muscles of denervated mice. The expression of FOXO1 increased in mice that had undergone denervation. Cathepsin L was also increased in denervated mice. Denervation significantly reduced the expression of PGC‐1β and MCAD. Quantitative real‐time RT‐PCR data from control samples were set at 100 arbitrary units. Each value is presented as the mean ± SE (control: *N* = 3, denervation: *N* = 4). Statistical analyses were performed using Student’s two‐tailed unpaired *t*‐test. ***P < 0.001, **P < 0.01, *P < 0.05 versus control.

We analyzed the expression of the PGC‐1β gene under other conditions with increased endogenous FOXO1 expression. For one of these conditions, we subjected the skeletal muscles of mice to denervation. After 12 days, we dissected the mice. The skeletal muscle (gastrocnemius) weights were 141 ± 6 mg (control, *N* = 3) and 82 ± 2 mg (denervation, *N* = 4; *P* < 0.001), showing muscle atrophy associated with the denervation. Increased FOXO1 as well as cathepsin L mRNA expression was also observed in the skeletal muscles with denervation (Fig. 2). We also observed increased FOXO1 protein levels in skeletal muscles with denervation (data not shown). A marked decrease of PGC‐1β expression, as well as MCAD expression, was observed in the skeletal muscles with denervation (Fig. 2). Denervation increased 36B4 (reference gene) expression. We used another reference gene (18S), whose expression was not increased by denervation, and observed significant decrease of PGC‐1β expression, as well as MCAD expression (Fig. 2).

**Fig. 3:**
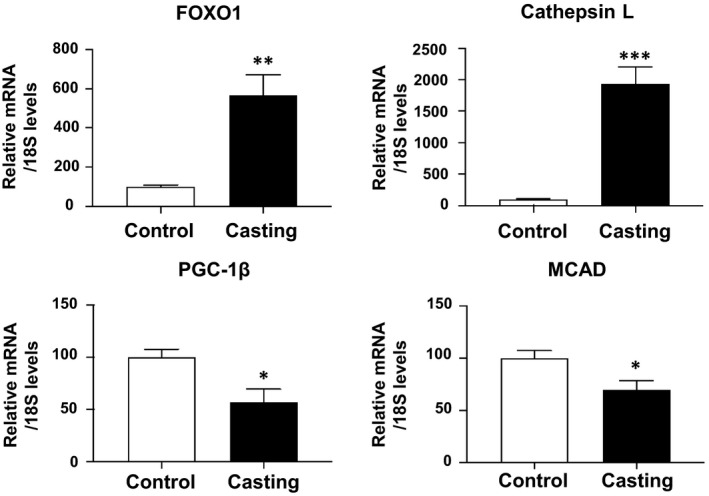
Gene expression analysis of FOXO1, cathepsin L, PGC‐1β, and MCAD in the skeletal muscles of plaster‐casted mice. The expression of FOXO1 and cathepsin L was increased upon unloading using a plaster cast. The expression of PGC‐1β and MCAD was decreased by plaster cast. Quantitative real‐time RT‐PCR data from control samples were set at 100 arbitrary units. Each value is presented as the mean ± SE (control: *N* = 5, casting: *N* = 4). Statistical analyses were performed using Student’s two‐tailed unpaired *t*‐test. ***P < 0.001, **P < 0.01, *P < 0.05 versus control.

Next, we used skeletal muscles subjected to unloading with a plaster cast. Unloading using a plaster cast for 11 days caused muscle atrophy. The skeletal muscle (gastrocnemius) weight was 150 ± 3 mg for the control group (*N* = 5) and 103 ± 5 mg for the group with a plaster cast (*N* = 4; *P* < 0.001). The plaster cast increased the mRNA expression of FOXO1 and its target cathepsin L, along with decreased PGC‐1β and MCAD expression (Fig. 3). Plaster cast also increased 36B4 (reference gene) expression. We used another reference gene (18S), whose expression was not increased by plaster cast, and observed significant decrease in PGC‐1β expression, as well as MCAD expression (Fig. 3).

**Fig. 4:**
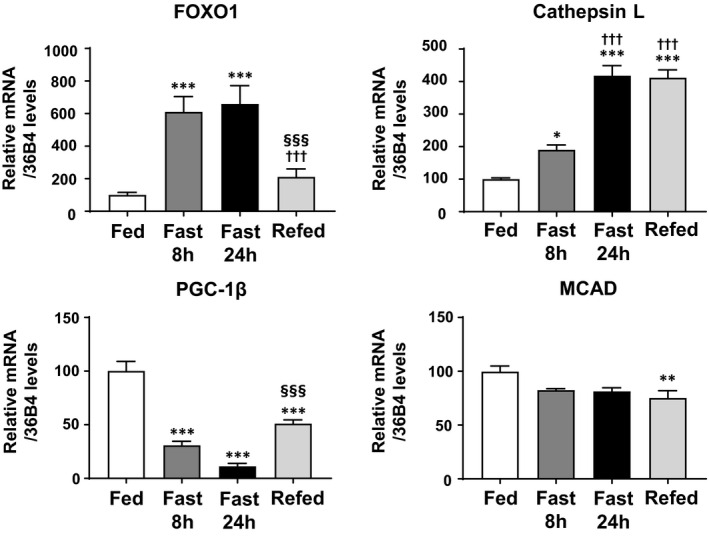
Gene expression analysis of FOXO1, cathepsin L, PGC‐1β, and MCAD in the skeletal muscles of fasted and refed mice. Fasting for 8 and 24 h increased the expression of FOXO1 and cathepsin L. Subsequent refeeding reduced FOXO1 expression. In contrast, the expression of PGC‐1β was decreased by fasting for 8 and 24 h, and the expression was recovered by refeeding. The MCAD expression was not markedly changed. Quantitative real‐time RT‐PCR data from fed samples were set at 100 arbitrary units. Each value is presented as the mean ± SE (*N* = 6). Statistical analyses were performed using one‐way analysis of variance followed by Tukey’s *post hoc* test. ***P < 0.001, **P < 0.01, *P < 0.05 versus fed; ^†††^ *P* < 0.001 versus fast for 8 h; ^§§§^ *P* < 0.001 versus fast for 24 h.

We also attempted to apply another condition with changed FOXO1 expression: fasting and refeeding. Fasting for 8 or 24 h increased FOXO1 expression in skeletal muscles. Fasting for 24 h followed by refeeding for 4 h downregulated the FOXO1 mRNA expression. Previously, we confirmed increased endogenous FOXO1 protein levels after 24‐h fasting [5]. Cathepsin L expression was gradually increased by fasting for 8 and 24 h, but it was not decreased by refeeding for 4 h. Therefore, cathepsin L mRNA may be stable against degradation for this period. PGC‐1β expression was gradually decreased by fasting for 8 and 24 h. Interestingly, refeeding for 4 h after fasting for 24 h recovered the PGC‐1β expression, compared with that upon fasting for 24 h alone (Fig. 4). MCAD expression was slightly decreased by fasting (8 or 24 h) and not markedly changed by refeeding (Fig. 4). Taking these findings together, in the skeletal muscles of mice, an inverse correlation was observed between FOXO1 and PGC‐1β (Figs 1, 2, 3, 4), suggesting that PGC‐1β expression is negatively regulated by FOXO1.

### Decreased PGC‐1β expression and decreased markers of mitochondrial density

**Fig. 5:**
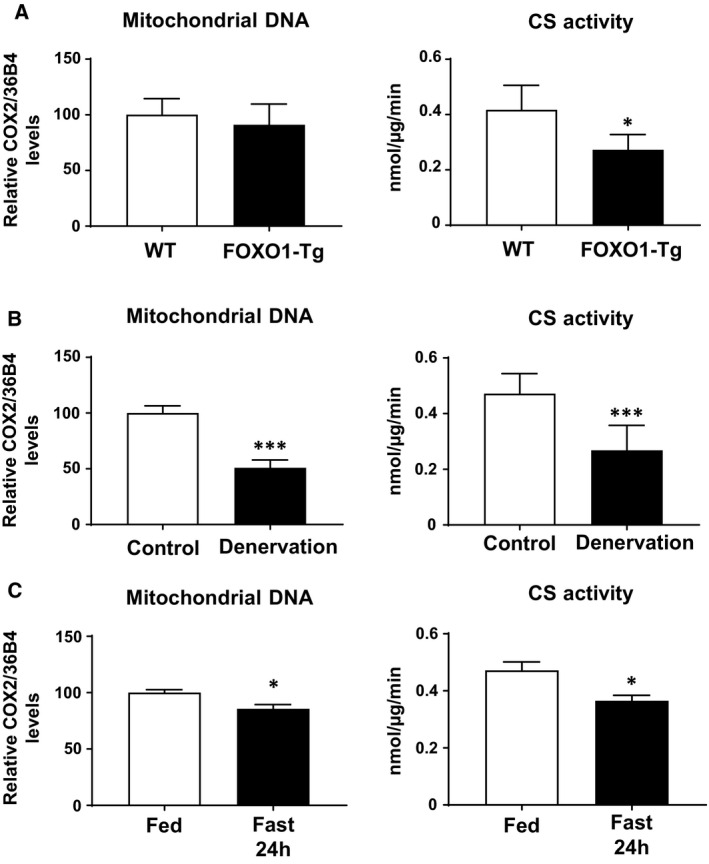
mtDNA content and CS activity in skeletal muscle. mtDNA content and CS activity in (A). Skeletal muscles of FOXO1‐overexpressing mice. The skeletal muscle weight of the wild‐type control was 127 ± 6 mg (*N* = 5) and that of the FOXO1‐Tg mice was 97 ± 5 mg (*N* = 5; *P* < 0.01). (B) Skeletal muscles of mice with denervation. The skeletal muscle weights were 138 ± 3 mg (control, *N* = 6) and 90 ± 2 mg (denervation, *N* = 8; *P* < 0.001). (C) Skeletal muscles of mice with fasting 24 h. The skeletal muscle weights were 138 ± 3 mg (control, *N* = 6) and 119 ± 2 mg (fasting 24 h, *N* = 6; *P* < 0.001). Each value is presented as the mean ± SE. Statistical analyses were performed using Student’s two‐tailed unpaired *t*‐test. ***P < 0.001, *P < 0.05 versus respective control.

PGC‐1β is known to increase mitochondrial content [12]; therefore, we examined mtDNA levels and mitochondrial enzyme CS activity as markers of mitochondrial density. Mitochondrially encoded COX2 (*Cox2*) DNA levels were slightly decreased, and CS activity was also significantly decreased in FOXO1‐Tg mice (Fig. 5A). In addition, decreased mtDNA levels and decreased CS activity were observed after denervation (Fig. 5B). Moreover, fasting for 24 h caused decreased mtDNA level and decreased CS activity (Fig. 5C). Thus, decreased PGC‐1β mRNA levels caused by FOXO1 appeared to lead to decreased functional PGC‐1β protein expression, concomitant with decreased mitochondrial content.

### Attenuation of decreased PGC‐1β expression by fasting in the skeletal muscles of FOXO1‐KO mice

**Fig. 6:**
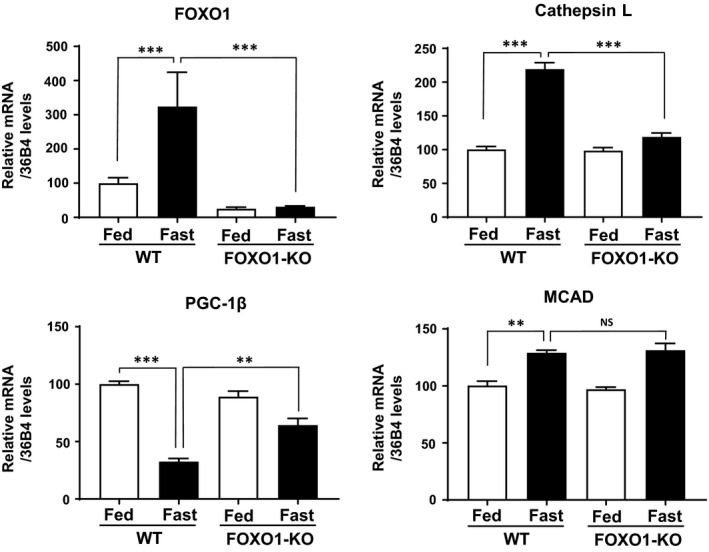
Gene expression analysis of the skeletal muscles of FOXO1‐KO mice. Gene expression in skeletal muscle of fed and fasted FOXO1‐KO mice. FOXO1‐KO and wild‐type mice were either allowed *ad libitum* access to food or subjected to a 24‐h fast (wild‐type fed, *n* = 3; wild‐type fasted, *n* = 4; KO fed, *n* = 4; KO fasted, *n* = 4). Expression levels of FOXO1, cathepsin L, PGC‐1β, and MCAD in skeletal muscle are shown. Quantitative real‐time RT‐PCR data from fed wild‐type mice were set at 100 arbitrary units. Each value is presented as the mean ± SE. Statistical analyses were performed using two‐way analysis of variance followed by Tukey’s *post hoc* test. ***P < 0.001, **P < 0.01. NS, not significant.

For loss‐of‐function experiments, we used skeletal muscle‐specific FOXO1‐KO mice [5]. In wild‐type control mice, fasting caused increased FOXO1 mRNA levels concomitant with increased cathepsin L mRNA (Fig. 6). In FOXO1‐KO mice, FOXO1 mRNA levels were very low in both fed and fasting samples, as expected. In a previous study, we confirmed diminished endogenous FOXO1 protein levels in the skeletal muscle of FOXO1‐KO mice in fed and fasting conditions [5]. In FOXO1‐KO mice, fasting‐induced cathepsin L mRNA expression was significantly attenuated (Fig. 6). In wild‐type mice, PGC‐1β mRNA levels were decreased by fasting. On the other hand, the fasting‐induced PGC‐1β mRNA decrease was significantly attenuated in FOXO1‐KO mice. The data indicated that fasting‐caused PGC‐1β mRNA decrease was likely to be mediated by FOXO1.

### PGC‐1β gene expression change induced by FOXO1 activation in C2C12 cells

**Fig. 7:**
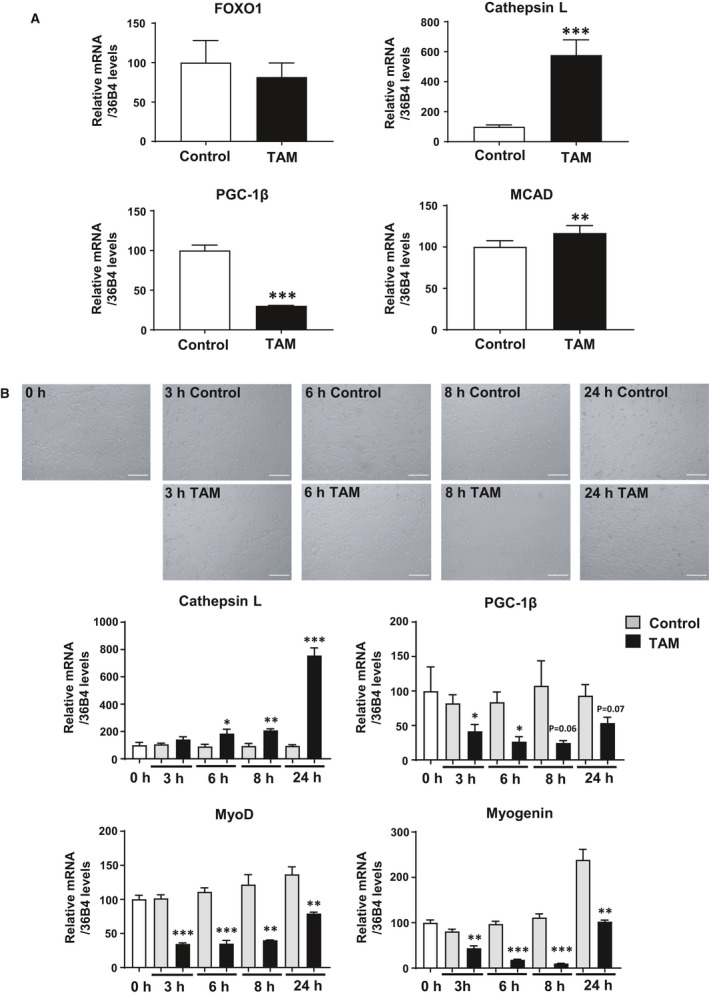
Effect of FOXO1 activation on the expression of PGC‐1β in C2C12 cells. (A) Tamoxifen (TAM) was added to FOXO1‐ER cells, and 24 h later, mRNA expression was analyzed. Expression levels of FOXO1, cathepsin L, PGC‐1β, and MCAD are shown. Quantitative real‐time RT‐PCR data from controls were set at 100 arbitrary units. Each value is presented as the mean ± SE (*N* = 6). Statistical analyses were performed using Student’s two‐tailed unpaired *t*‐test. ***P < 0.001, **P < 0.01 versus control. (B) Time course (3, 6, 8, and 24 h) after tamoxifen treatment; microscopic views were observed, and mRNA expression levels were analyzed. Scale bars, 100 µm. Quantitative real‐time RT‐PCR data from 0 h were set at 100 arbitrary units. Each value is presented as the mean ± SE (*N* = 4). Statistical analyses were performed using Student’s two‐tailed unpaired *t*‐test. ***P < 0.001, **P < 0.01, *P < 0.05 versus control (vehicle).

In order to understand the causal relationship between FOXO1 expression and PGC‐1β expression, we used a tamoxifen‐inducible FOXO1 activation system in C2C12 myoblast cells. Namely, tamoxifen treatment induces the translocation of FOXO1‐ER fusion protein (FOXO1‐ER) from the cytoplasm to the nucleus and causes FOXO1‐mediated target gene activation [4, 23]. In the presence of tamoxifen (for 24 h), FOXO1 mRNA expression remained unchanged (Fig. 7A), which is consistent with the findings of a previous study [4]. Cathepsin L expression was increased by tamoxifen treatment, indicating FOXO1 activation. Interestingly, in the presence of tamoxifen (FOXO1 activation), there was a marked decrease of PGC‐1β expression (Fig. 7A). Thus, PGC‐1β gene expression was negatively regulated by FOXO1 in C2C12 myoblast cells. In this experiment, MCAD expression was slightly increased by tamoxifen treatment.

Forkhead box protein O1 was reported to suppress muscle cell differentiation [1, 25, 26]; therefore, we examined the change in muscle differentiation marker gene expression during this experimental period. We examined the time course of PGC‐1β and differentiation marker gene expression. C2C12 cells expressing FOXO1‐ER were treated with tamoxifen, and at 3, 6, 8, and 24 h after treatment, mRNA expression levels were examined. Three hours after treatment, PGC‐1β and MyoD and myogenin (differentiation marker genes) mRNA levels were decreased (Fig. 7B). We also observed microscopic views of cells; there were no marked phenotypical changes between the vehicle (control) and tamoxifen treatment groups during this time period (Fig. 7B). We used cells without differentiation stimuli (not using differentiation medium) in confluent cells. Thus, we consider that the cells did not differentiate in these conditions. Thus, the decreased PGC‐1β mRNA levels were not likely to be caused by decreased differentiation (not a result of the differentiation process), but by direct suppression by FOXO1.

### FOXO1 expression and PGC‐1α expression changes

**Fig. 8:**
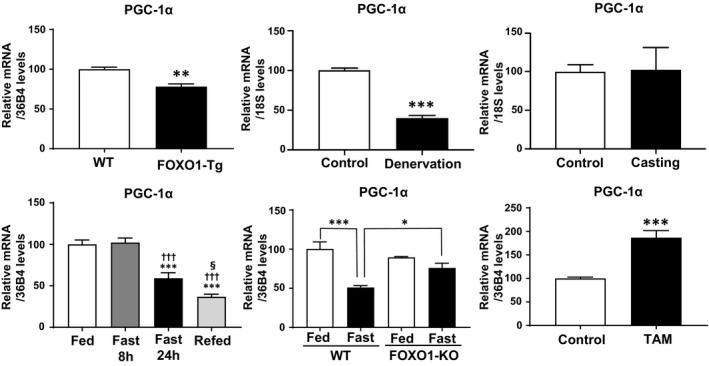
Gene expression of PGC‐1α in skeletal muscles and cells. The expression of PGC‐1α was examined in the samples used in Figs 1, 2, 3, 4, 6, and 7A. Each value is presented as the mean ± SE. For FOXO1‐Tg experiment, statistical analyses were performed using Student’s two‐tailed unpaired *t*‐test (*N* = 5). **P < 0.01 versus wild‐type. For denervation experiment, statistical analyses were performed using Student’s two‐tailed unpaired *t*‐test (control: *N* = 3, denervation: *N* = 4). ***P < 0.001 versus control. For casting experiment, statistical analyses were performed using Student’s two‐tailed unpaired *t*‐test (control: *N* = 5, casting: *N* = 4). For fasting experiment, statistical analyses were performed using one‐way analysis of variance followed by Tukey’s *post hoc* test (*N* = 6). ***P < 0.001 versus fed; ^†††^ *P* < 0.001 versus fast for 8 h; ^§^ *P* < 0.05 versus fast for 24 h. For FOXO1‐KO experiment, statistical analyses were performed using two‐way analysis of variance followed by Tukey’s *post hoc* test (wild‐type fed, *n* = 3; wild‐type fasted, *n* = 4; KO fed, *n* = 4; KO fasted, *n* = 4). ***P < 0.001, *P < 0.05. For FOXO1‐ER experiment, statistical analyses were performed using Student’s two‐tailed unpaired *t*‐test (*N* = 6). ***P < 0.001 versus control.

For comparison, we also examined PGC‐1α (PGC‐1β homologue) expression in the samples used for Figs 1, 2, 3, 4, 6, and 7 (Fig. 8). PGC‐1α expression was decreased in FOXO1‐Tg mice and in those subjected to denervation, but not unloading with a plaster cast, while PGC‐1β expression was decreased in these groups. Meanwhile, upon fasting for 8 h, PGC‐1α expression did not change; however, upon fasting for 24 h followed by refeeding, PGC‐1α expression was decreased (Fig. 8). In FOXO1‐KO mice, fasting (24 h) caused decrease of PGC‐1α expression was attenuated (Fig. 8). In the case of the tamoxifen‐activated FOXO1‐ER experiment, PGC‐1α expression was not decreased but rather significantly increased (Fig. 8). Thus, PGC‐1α expression appears not to be simply downregulated by FOXO1 activation. PGC‐1α is also known to increase MCAD expression [11]. Thus, the increased MCAD level observed in the FOXO1‐ER cells (Fig. 7A) may be explained by the increased PGC‐1α expression, but other possibilities should also be considered.

### Possible physiological significance and mechanism of FOXO1‐mediated suppressed PGC‐1β expression

In this study, we observed that the activation of FOXO1 suppressed PGC‐1β expression in skeletal muscles and myoblast cells. We obtained a clue regarding the mechanism of PGC‐1β gene regulation, which was previously largely unknown. FOXO1 activation causes skeletal muscle atrophy [3], and PGC‐1β activation causes increased energy expenditure [10]. During atrophy with FOXO1 activation, decreased PGC‐1β with decreased energy expenditure appears to be physiologically reasonable, to avoid wasting energy in order to prevent a greater decrease of muscle mass.

Forkhead box protein O1 has been reported to increase the degradation of mitochondria, leading to a decrease in mitochondrial content [27]. As described in the Introduction, PGC‐1β increases mitochondrial content [12]. Thus, FOXO1 caused downregulation of PGC‐1β as described in this study, which is consistent with decreased mitochondrial content. Indeed, in FOXO1‐Tg mice, the amount of red muscle fiber, which is rich in mitochondria, is decreased [3]. Additionally, the skeletal muscle of mice with plaster cast or denervation shows a decreased red muscle fiber level, that is, decreased mitochondria concomitant with increased FOXO1 expression [3, 18]. Thus, decreased mitochondrial content with increased FOXO1 expression may be mediated by FOXO1‐induced PGC‐1β suppression.

**Fig. 9:**
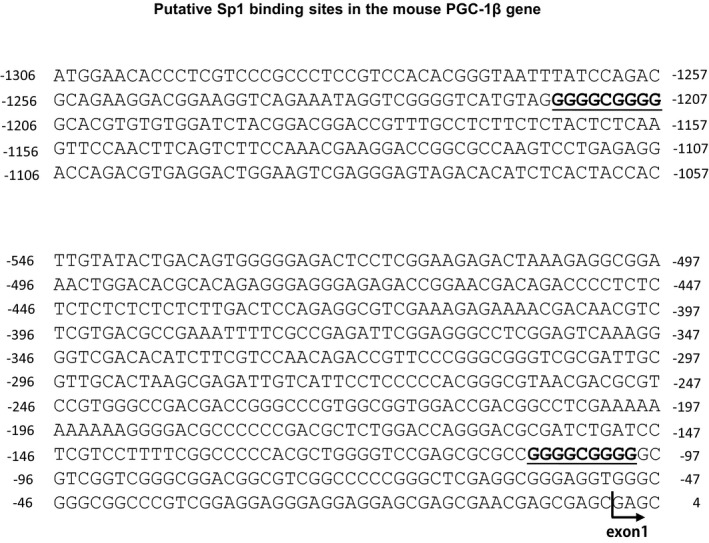
Putative Sp1 binding sites (GGGGCGGGG) in the promoter of the mouse PGC‐1β gene. Upstream of the PGC‐1β gene from +4 to −546 and −1057 to −1306 is shown. The transcription start site is counted as +1. The Sp1 binding sites (GGGGCGGGG) are underlined (−99 to −107 and −1207 to −1215).

Meanwhile, how FOXO1 downregulates the PGC‐1β gene is currently unclear. FOXO1 binds to the genomic DNA sequence, with the Daf16 binding element (DBE) (consensus: TT[G/A]TTTAC) [28] or insulin response element (IRE; consensus: TT[G/A]TTTTG) [29]. However, there were no consensus DBE or IRE up to 2 kb upstream from the transcription start site. Meanwhile, FOXO1 has been reported to physically interact with other transcription factors, such as nuclear receptors, and to positively and negatively regulate target gene expression [30, 31, 32]. However, there were no typical nuclear receptor binding sites, such as glucocorticoid (atrophic hormone) receptor response elements (GREs; consensus: AGAACA), up to 2 kb upstream from the transcription site. Meanwhile, Yasui *et al*. [8] reported Sp1 binding sites are involved in FOXO1‐mediated repression of musclin gene expression. Notably, there are two putative Sp1 binding sites (consensus: GGGGCGGGG) [33] in the mouse PGC‐1β gene at 0.1 and 1.2 kb upstream from the transcription start site (Fig. 9). Moreover, Shintaku *et al*. [34] showed transcription factors MyoD and RelB bind within the first intron of the PGC‐1β gene and activate transcription. FOXO1 may regulate the PGC‐1β gene expression by directly binding to the PGC‐1β promoter, or by interacting with other transcription regulators (such as SP1, MyoD, and RelB) binding to the PGC‐1β promoter. On the other hand, microarray data showed decreased MyoD expression in the skeletal muscles of FOXO1‐Tg mice, compared with that in wild‐type control mice (0.43‐fold; Table 2). Additionally, we observed decreased MyoD levels in C2C12 cells expressing FOXO1‐ER using tamoxifen treatment (Fig. 7B). FOXO1 may suppress PGC‐1β gene expression via suppressing MyoD expression. Further work is required to clarify this issue.

## Conflict of interest

The authors declare no conflict of interest.

## Author contributions

SN and YH analyzed the data and undertook the statistical analyses. MO and YH performed cell experiment and collected the data. SA and SM performed enzyme assays and mtDNA experiments. TK performed FOXO1‐KO experiments. YK prepared the manuscript. All authors reviewed the results and approved the final version of the manuscript.

## Data Availability

The microarray data were submitted to the GEO database (https://www.ncbi.nlm.nih.gov/geo/). The records have been assigned GEO accession numbers as GSE146919.
